# Case Report: Subtotal Hemispherotomy Modulates the Epileptic Spasms in Aicardi Syndrome

**DOI:** 10.3389/fneur.2021.683729

**Published:** 2021-06-24

**Authors:** Yasushi Iimura, Hidenori Sugano, Takumi Mitsuhashi, Tetsuya Ueda, Kostadin Karagiozov, Shimpei Abe, Hiroshi Otsubo

**Affiliations:** ^1^Department of Neurosurgery, Epilepsy Center, Juntendo University, Tokyo, Japan; ^2^Department of Pediatrics, Epilepsy Center, Juntendo University, Tokyo, Japan; ^3^Division of Neurology, The Hospital for Sick Children, Toronto, ON, Canada

**Keywords:** epileptic spasms, high frequency oscillations, phase-amplitude coupling, subtotal hemispherotomy, modulation index

## Abstract

The mechanism of epileptic spasms (ES) in Aicardi syndrome (AS) remains obscure. We compared intraoperative high-frequency oscillations (HFOs) and phase-amplitude coupling (PAC) before and after subtotal hemispherotomy in a 3-month-old girl with drug-resistant ES secondary to AS. Fetal ultrasonography showing corpus callosum agenesis, bilateral ventricular dilatation, and a large choroid plexus cyst confirmed AS diagnosis. Her ES started when she was 1 month old and had ten series of clustered ES per day despite phenobarbital and vitamin B6 treatment. After subtotal hemispherotomy, her ES dramatically improved. We analyzed two intraoperative electrocorticography modalities: (1), occurrence rate (OR) of HFOs; (2), PAC of HFOs and slow wave bands in the frontal, central, and parietal areas. We hypothesized that HFOs and PAC could be the biomarkers for efficacy of subtotal hemispherotomy in AS with ES. PAC in all three areas and OR of HFOs in the frontal and parietal areas significantly decreased, while OR of HFOs in the central area remained unchanged after subtotal hemispherotomy. We have demonstrated the usefulness of evaluating intraoperative HFOs and PAC to assess subtotal hemispherotomy effectiveness in AS patients with ES. Disconnecting the thalamocortical and subcortical pathways in the epileptic network plays a role in controlling ES generation.

## Introduction

Aicardi syndrome (AS) is a neurodevelopmental disorder characterized by seizures and agenesis of the corpus callosum and chorioretinal lacunae (1). Children with AS have psychomotor retardation and poor functional outcomes (2). Epileptic spasms (ES) are the most typical seizure type in AS, becoming drug-resistant with time (3). The therapeutic purpose in children with AS is to control the ES and improve the functional outcome.

Reported medical treatments for ESs in general include adrenocorticotropic hormone (ACTH), vigabatrin, ketogenic diet, and various antiepileptic drugs, but the seizure outcome is unsatisfactory (4). Seizure control was also unsatisfactory following lesionectomy, hemispherectomy, multilobar resection, and vagus nerve stimulation (VNS) as a surgical treatment for the ES (3–6). Chugani et al. reported that subtotal hemispherectomy effectively controlled ES, however, this procedure has not been reported in patients with AS (4, 7).

Clinically, the mechanism of ES has not been determined yet, so it is categorized into focal, generalized, or unknown onset based on the seizure type (8). Several electrophysiological analyses, including high-frequency oscillations (HFOs) and phase-amplitude coupling (PAC), were used to reveal the mechanism of ES. HFOs have become a promising biomarker for the epileptogenic zones in invasive and non-invasive EEG (9–11). PAC of HFOs and slow wave bands, rather than HFOs alone, was applied to localize the epileptic foci (12). Children with ES were reported to demonstrate high occurrence rate (OR) of HFOs and high values of PAC (13, 14).

We describe a case of an AS patient without hemiparesis who developed drug-resistant ES and in whom we performed subtotal hemispherotomy. Intraoperative electrocorticography (ECoG) was performed, and HFOs and PAC were analyzed before and after surgery as an assessment of subtotal hemispherotomy efficacy. To the best of our knowledge, this is the first report of intraoperative ECoG recording and quantitative analysis in an AS patient with ES. We hypothesized that intraoperative HFOs and PAC could act as biomarkers for ES network disconnection.

## Case Description

### History and Examinations

The female fetus was diagnosed with AS by ultrasonography (US) at 30 weeks of gestation. The US showed corpus callosum agenesis, bilateral ventricle dilatation, and a large choroid plexus cyst in the left trigone of the lateral ventricle. The baby was born without any complications at 41 weeks of gestation at 3,128 g. She had no family history of epileptic disorders, her muscle tone was normal, and there was no paresis. Ophthalmologic examination revealed bilateral chorioretinal lacunae.

**Figure 1:**
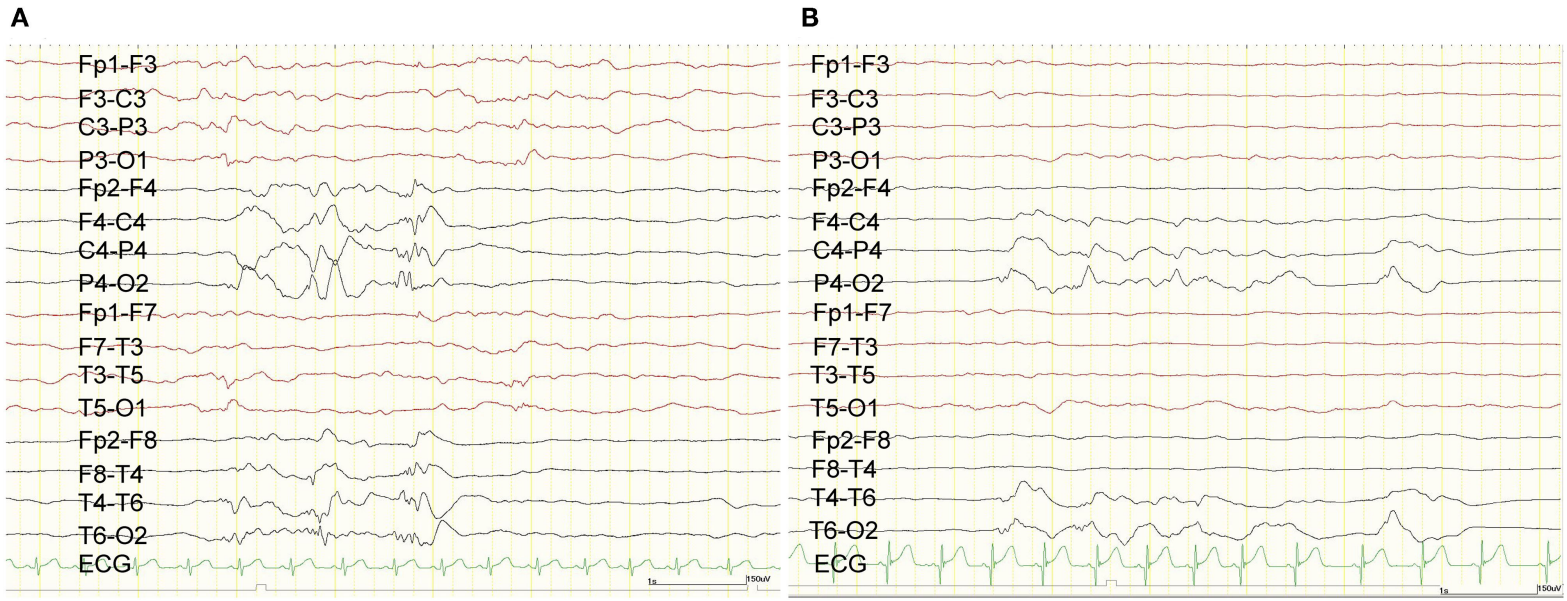
Scalp EEG. Preoperative scalp EEG revealed interictal epileptic discharges originating from the right hemisphere **(A)**. Postoperative scalp EEG demonstrated that the interictal epileptic discharges from the right hemisphere were reduced **(B)**.

**Figure 2:**
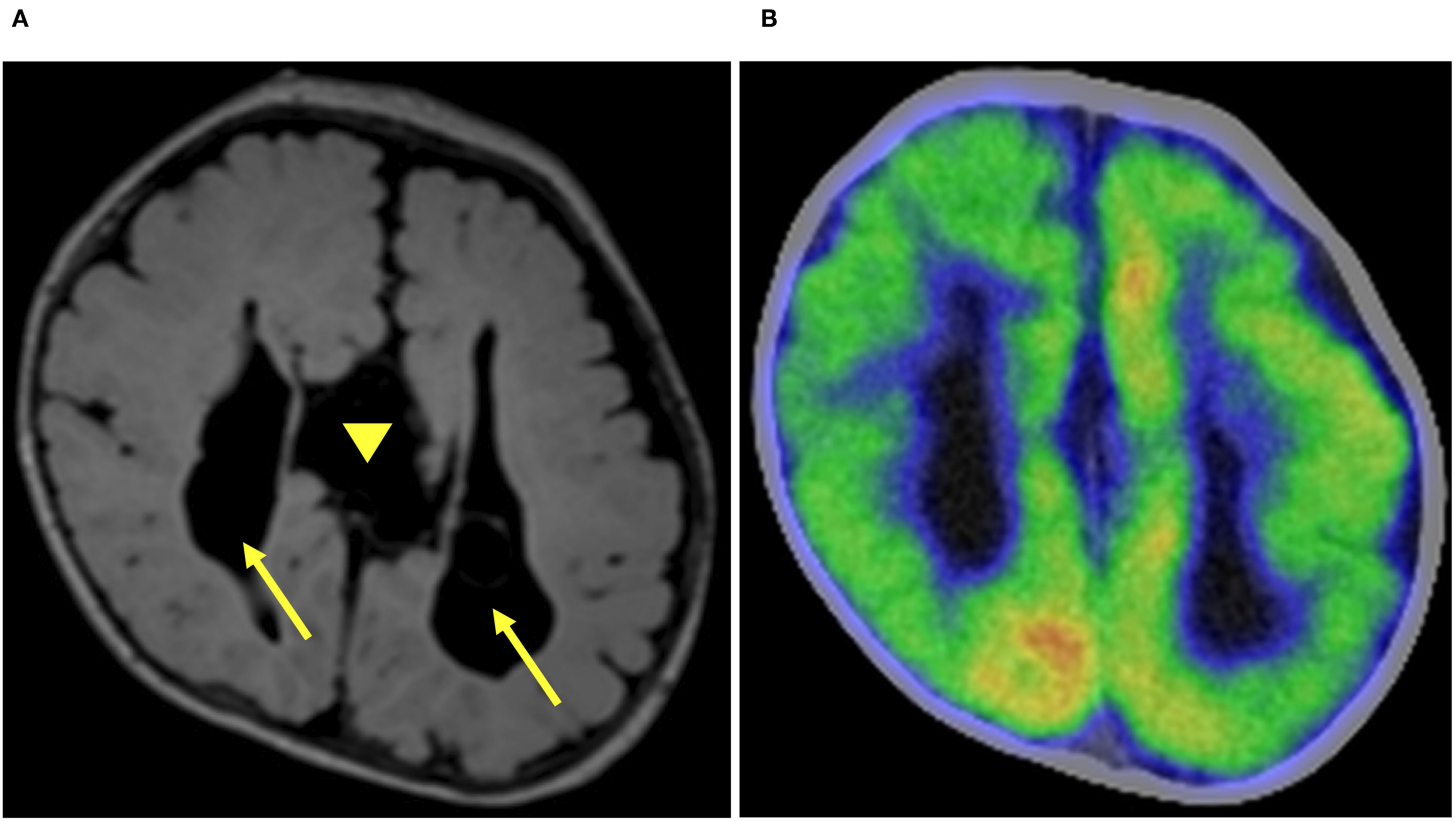
Preoperative imaging. Preoperative axial fluid-attenuated inversion recovery magnetic resonance imaging (FLAIR MRI) demonstrated corpus callosum agenesis (arrow head) and bilateral (right dominant) ventricular dilatation (arrow) **(A)**. Fluorodeoxyglucose-positron emission tomography-computed tomography showed hypometabolism in the right hemisphere **(B)**.

The ES started when she was 1 month old and escalated to ten series of clustered ES per day despite treatment with phenobarbital and vitamin B6. The semiology of her ES was symmetric. Prolonged scalp video interictal EEG showed that multifocal epileptiform abnormalities were generated only over the right hemisphere (Figure 1A). Interictal background EEG in the left hemisphere was grossly normal. Ictal EEG findings demonstrated that high amplitude positive slow waves were originated from the right hemisphere. Electromyogram showed as a rhombus shape, consistent with ES. Magnetic resonance imaging (MRI) demonstrated corpus callosum agenesis, bilateral ventricle dilatation with right-side predominance, and a large choroid plexus cyst in the left trigone of the lateral ventricle (Figure 2A). Fluorodeoxyglucose positron emission tomography- computed tomography (PET-CT) showed hypometabolism in the right hemisphere (Figure 2B). EEG and PET-CT findings were lateralized, suggesting that epilepsy surgery may improve the seizure outcome. In addition, due to the absence of hemiparesis, we performed the right subtotal hemispherotomy at the age of 3 months based on the results of the multidisciplinary consensus conference. At the time of surgery, her head control was unstable, but she had no hemiparesis.

### Surgical Procedure and Postoperative Seizure Outcome

**Figure 3:**
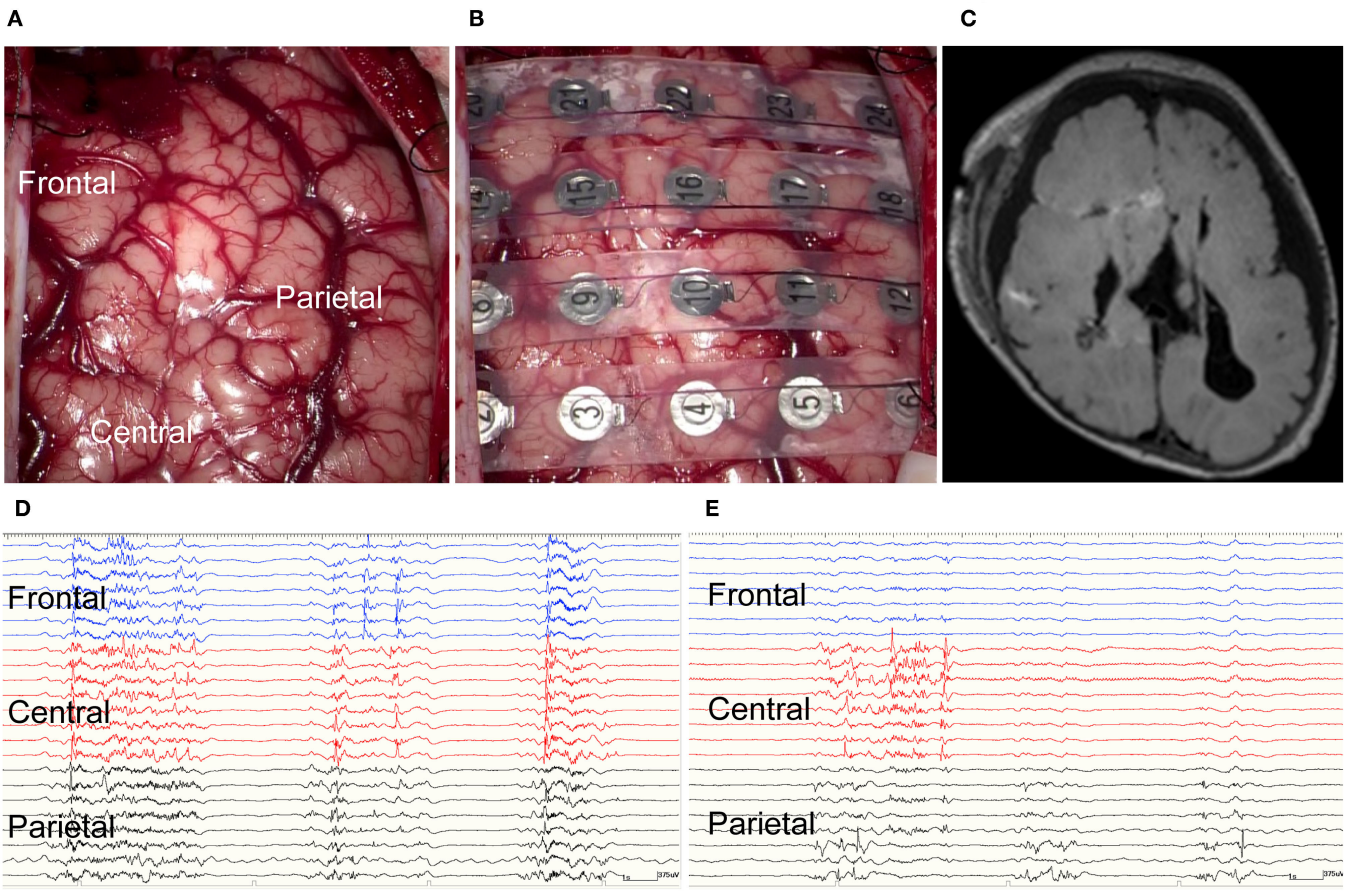
Intraoperative electrocorticography and postoperative imaging. **(A)** Intraoperative findings included absent Roland vein and ambiguous central sulcus. The sclerotic cortex was identified in the central area. **(B)** Intraoperative electrocorticography. **(C)** Postoperative axial fluid-attenuated inversion recovery magnetic resonance imaging (FLAIR MRI) demonstrated the disconnection line. **(D)** Intraoperative electrocorticography before subtotal hemispherotomy. **(E)** Intraoperative electrocorticography after subtotal hemispherotomy.

The patient's head was fastened on a horseshoe-shaped headrest at 45 degrees rotation to the opposite side of the craniotomy. We used a neuronavigation system (StealthStation surgical navigation system cranial application, Version 5; Medtronic, Minneapolis, MN, USA) to decide on the disconnection line and simulate it over the scalp before incising the skin. Subsequently, we performed a craniotomy with a curvilinear scalp incision, based on our preliminary design, to expose an adequate surgical field. After the craniotomy, we opened the Sylvian fissure and identified the limen insulae. The inferior horn of the lateral ventricle was opened through the inferior periinsular sulcus. The fornix and tail of the hippocampus were disconnected until the ambient cistern was visualized. These steps completely disconnected between the temporal lobe structures and basal ganglia. Parietal disconnection was performed along with the postcentral sulcus. Operative findings indicated that the Roland vein was absent, and the central sulcus was ambiguous (Figure 3A). The sclerotic cortex was identified in the central area. Corpus callosotomy was not done due to corpus callosum agenesis. Subsequently, we performed frontal disconnection along the precentral sulcus. Subtotal hemispherotomy was performed, sparing the motor cortex (Figure 3C).

The patient experienced no complications during or after the subtotal hemispherotomy. The postoperative ES frequency has decreased to three series per day. Postoperative interictal scalp EEG showed that the multifocal epileptiform abnormalities were originated from the right central and posterior quadrant region. Ictal EEG findings demonstrated that high amplitude positive slow waves were originated from the right hemisphere same as before subtotal hemispherotomy. Both frequency and amplitude during interictal and ictal epileptic discharges have decreased (Figure 1B). We administered ACTH therapy (0.015 mg/kg/day) for 2 weeks at the age of 4.6 months. Despite phenobarbital (90 mg/day), valproic acid (400 mg/day), and perampanel (1 mg/day) with no adverse events, the three daily ES series remained even 19 months after the surgery. She had psychomotor developmental delay despite of the absence of hemiparesis.

### HFOs and PAC Analysis in the Intraoperative ECoG

We used NeuroFax (Nihon-Koden, Tokyo, Japan) at a sampling rate of 2,000 Hz to record intraoperative ECoG under total propofol-based intravenous anesthesia. The recording was done before and after performing the subtotal hemispherotomy and lasted 10 min at each time point (Figures 3D,E). We placed a 4 × 6 subdural grid (Unique Medical Co., Ltd., Tokyo, Japan), consisting of 24 platinum electrodes (4-mm diameter and 10-mm distance), from the frontal to the parietal lobes (Figure 3B). The electrodes were placed over the frontal, central, and parietal areas, *n* = 8 in each.

HFOs on the bipolar montage were automatically detected by MATLAB same as previous report (10). A band-pass filter at 80–200 Hz and a high-pass filter at 200 Hz were used to extract ripples (80–200 Hz) and fast ripples (FRs; 200–300 Hz), respectively. Because the interictal HFOs appear as intermittent peaks in the envelope curve, the envelope curve of the filtered ECoG was calculated by Hilbert transform. Events of ripples and FRs are detected by thresholding. Epochs with the envelope curve exceeding the threshold are detected as events of ripples and FRs, respectively. We visually inspected each ECoG epoch with a high-pass filter at 0.5 Hz and at 200 Hz to ensure that they were not contaminated by significant artifacts, such as environmental artifacts and muscle artifacts.

We defined the occurrence rate (OR) as the index of the HFOs. We calculated the OR of ripples and FRs after dividing each of the 10-min intraoperative ECoG recordings into ten 1-min epochs, and acquired ten OR of HFOs values for each of the three brain areas, before and after subtotal hemispherotomy.

The modulation index (MI) reflects the PAC degree of strength (13). It was calculated on the monopolar montage for each area using the EEGLAB, Phase-Amplitude Coupling Toolbox (PACT), v.0.17 (13). We analyzed the MI between HFOs (ripples and FRs) and the slow wave bands (0.5–4 Hz). We acquired ten MI values (HFOs and slow wave bands) in each area by analyzing the ten intraoperative ECoG epochs. We then compared the OR of HFOs and MI values in each area before and after subtotal hemispherotomy. All statistical analyses were performed using IBM SPSS Statistics for Windows, Version 25.0 (IBM Corp., Armonk, NY, USA). We compared the two groups by the Mann-Whitney *U*-test. Pair-wise comparisons were made by the Steel-Dwass test after testing for data normality with the *F*-test. Statistical significance was set at *p* < 0.05.

## Results of the HFOs and PAC Analysis in the Intraoperative ECoG

### HFO

#### OR of HFOs in Each Area Before Subtotal Hemispherotomy

**Figure 4:**
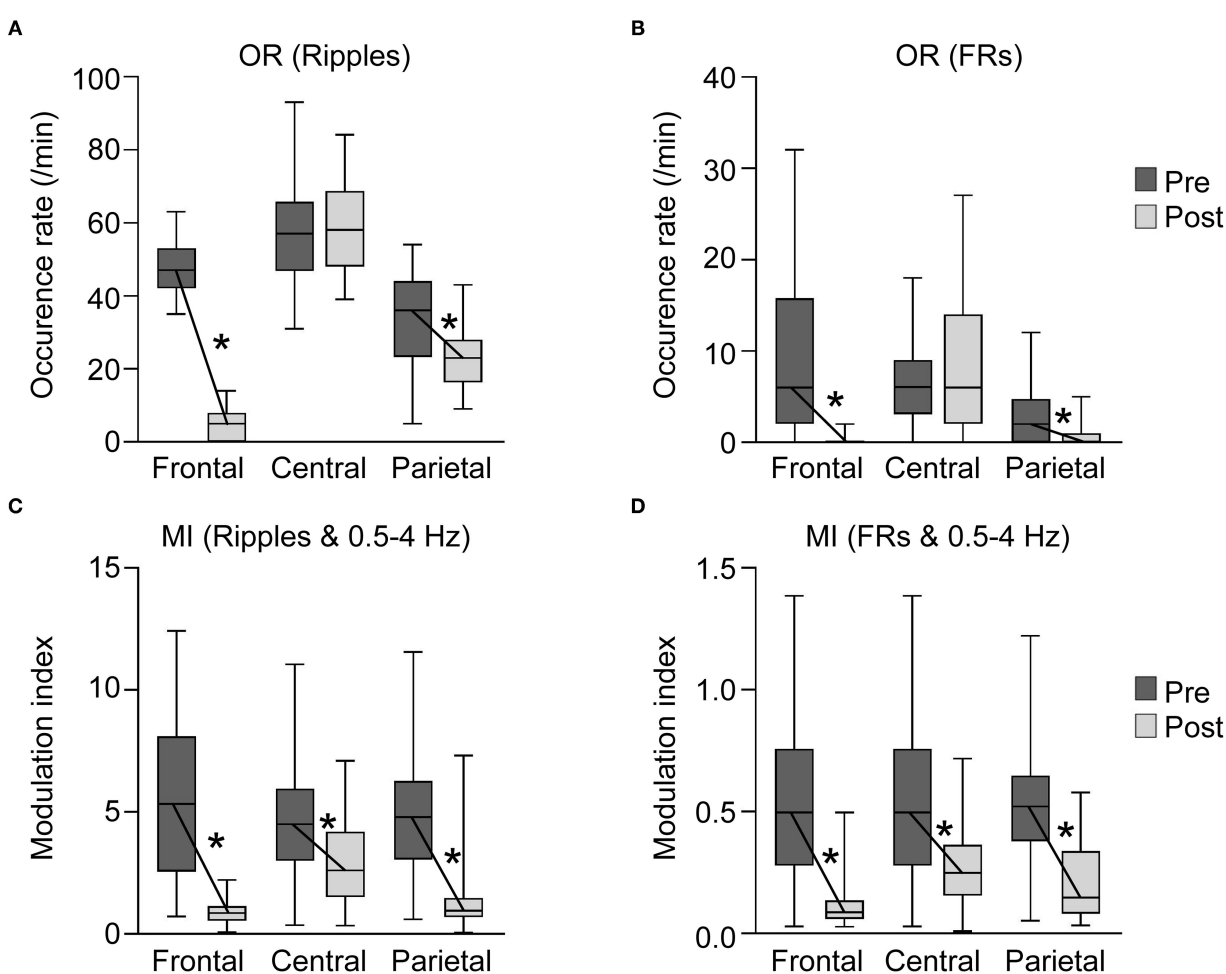
Occurrence rates (ORs) of high-frequency oscillations (HFO) and modulation index (MI) in each area before and after subtotal hemispherotomy. **(A,B)** OR of HFOs in the frontal and parietal areas decreased significantly after subtotal hemispherotomy (*p* < 0.01 for all), while they remained unchanged in the central area. **(C,D)** MI (HFO and slow wave bands) in all three areas decreased significantly after subtotal hemispherotomy (*p* < 0.01 for all). Pre, before subtotal hemispherotomy; Post, after subtotal hemispherotomy; *indicates *p* < 0.01.

OR of the ripples in the frontal, central, and parietal areas before subtotal hemispherotomy were 47.5 ± 7.7, 56.0 ± 13.3, and 33.3 ± 13.2, respectively (mean ± standard deviation) (Figure 4A). The OR of ripples in the central area was higher than that in the frontal and parietal areas, and that in the frontal area was higher than in the parietal area (*p* < 0.01 for all).

The OR of FRs in the frontal, central, and parietal areas before subtotal hemispherotomy were 9.7 ± 8.8, 6.6 ± 3.9, and 2.7 ± 2.8, respectively (Figure 4B). The OR of FRs in the frontal and central areas were higher than in the parietal area (*p* < 0.01 for both).

#### OR of HFOs in Each Area After Subtotal Hemispherotomy

The OR of ripples in the frontal, central, and parietal areas after subtotal hemispherotomy were 4.8 ± 3.9, 58.9 ± 11.4, and 23.2 ± 7.9, respectively, and those of FRs were 0.3 ± 0.6, 7.5 ± 6.7, and 0.8 ± 1.4, respectively. The OR of ripples and FRs in the central area remained higher than in the frontal and parietal areas (*p* < 0.01 for all).

#### Comparison OR of HFOs in Each Area Before and After Subtotal Hemispherotomy

The OR of ripples and FRs in the frontal and parietal areas decreased significantly after subtotal hemispherotomy (*p* < 0.01 for all), while they remained unchanged in the central area.

### PAC

#### MI in Each Area Before Subtotal Hemispherotomy

The MI in the frontal, central, and parietal areas before subtotal hemispherotomy were 5.6 ± 3.3, 4.7 ± 2.2, and 4.7 ± 2.1, respectively for ripples and slow wave bands, and 0.54 ± 0.32, 0.51 ± 0.23, and 0.52 ± 0.23, respectively, for FRs and slow wave bands (Figure 4C).

#### MI in Each Area After Subtotal Hemispherotomy

The MI in the frontal, central, and parietal areas after subtotal hemispherotomy were 0.9 ± 0.5, 2.9 ± 1.6, and 1.4 ± 1.5, respectively, for ripples and slow wave bands, and 0.11 ± 0.09, 0.28 ± 0.15, and 0.20 ± 0.15, respectively, for FRs and slow wave bands (Figure 4D). The MI of ripples or FRs and slow wave bands in the central area remained significantly higher than in the frontal and parietal areas (*p* < 0.01 for all).

#### Comparison of MI in Each Area Before and After Subtotal Hemispherotomy

MI of ripples or FRs and slow wave bands in all three areas significantly decreased after subtotal hemispherotomy (*p* < 0.01 for all).

## Discussion

### The Effectiveness of Subtotal Hemispherotomy for AS With ES

Previous surgical studies reported that hemispherectomy, multilobar resection, and VNS to treat ES in AS patients led to variable seizure outcomes (3, 6, 7). A hemispherectomy was performed in three AS patients with ES, and multilobar resection surgery was performed in two. Only one patient, who underwent hemispherectomy, became seizure-free. VNS was performed as a palliative surgery for AS patients with ES (3, 7, 15). Five of eleven children implanted with a VNS device in three previous studies showed some degree of seizure improvement (3, 7, 15). The contribution of surgical treatment for AS patients with ES has still been established.

In this study, we applied subtotal hemispherotomy to an AS patient with intractable ES. Subtotal hemispherectomy is effective in patients with ES with absent or mild hemiparesis (4, 5). In one study, ten of seventeen non-AS patients who underwent subtotal hemispherectomy became seizure-free (4). As our AS patient with intractable ES did not have hemiparesis, we applied subtotal hemispherotomy. The procedure successfully reduced the severity of ES. In addition, improvement in EEG findings suggest that subtotal hemispherotomy is effective for AS patients with ES.

Even in AS, where bilateral hemispheric abnormalities are often observed, epilepsy surgery may be considered when EEG and PET-CT findings are lateralized. Similarly, when EEG or PET-CT findings are lateralized after corpus callosotomy for ES, two-stage surgery, including subtotal hemispherotomy, may be applied. Earlier epilepsy surgery for ES is recommended to promote plasticity and psychomotor development (4). Although psychomotor development was not accelerated in this case, early surgical intervention for AS may be considered if it can be done safely.

Epilepsy surgery was expected to improve the seizure outcome because the lateralization of the epileptic focus was assumed by the EEG and PET-CT findings. We administered the ACTH therapy after performing the subtotal hemispherotomy. In general, only some patients with ES and without AS respond to ACTH therapy (16). In the presence of AS however, this therapy has been applied to a very limited number of cases, and remains controversial.

### HFOs and PAC Assessment During the Disconnection Surgery

#### HFOs

HFOs can be a physiological or pathological phenomenon (17). The resection of areas with high OR of HFOs led to good seizure control outcomes in children with ES (13). We documented intraoperative OR of HFOs in this AS patient, showing them to decrease significantly in the frontal and parietal areas after subtotal hemispherotomy. Thalamic inactivation was reported to reduce the occurrence of fast oscillations (18). Subtotal hemispherotomy includes disconnecting the thalamocortical pathway to the frontal and parietal areas. The decrease in HFOs in these areas after subtotal hemispherotomy indicated that they were pathological HFOs.

However, we cannot tell if the residual HFOs in the central area are physiological or pathological. The residual HFOs in the central area could be pathological as they were associated with the persisting ES. On the other hand, the ES had decreased to one-third of the preoperative frequency, and the patient showed no hemiparesis, suggesting that the residual HFOs in the central area could be physiological and originated in the motor cortex. We will need to investigate more patients undergoing subtotal hemispherotomy as a treatment for ES to verify whether the residual HFOs are physiological or pathological.

#### PAC

The resection of areas with high MI values led to a good seizure control in children with ES (13). MI could be a valuable biomarker for epileptogenic zones in ES. The post-disconnection MI of PAC decreased in our patient in all three areas, including the central area with its remaining HFOs.

The slow oscillations in the cortex originate from the corticothalamic system (18). High MI values might indicate that the slow oscillations originating from the thalamus play an important role in the generation of ES. Because subtotal hemispherotomy deafferentates thalamocortical pathway which are connecting thalamus and frontal, and thalamus and parietal areas, MI in frontal and parietal areas decreased. Moreover, disconnection of the subcortical pathway in the central area from frontal and parietal areas may have reduced the MI in the central area. Subcortical pathway may also play a role in the generation of slow oscillations. Hence, the MI decreased significantly in all three areas following subtotal hemispherotomy, consequently resulted in the improvement of ES.

These EEG analysis methods have limitations. A methodological problem in identifying interictal HFOs after filtering the signals is that “false” HFOs can be detected (19). It is necessary to carefully compare the data after filtering with the raw data. We have to pay attention to distinguish between false HFOs and true epileptic oscillations. PAC may falsely be identified using MI when both measured signals have a common driving source (20, 21). A modified MI is supposed to be robust to spurious PAC detections and may be worth investigating (22). We need to carefully analyze EEG and interpret the results, keeping in mind the limitations of these methods and the possible occurrences of “false” HFOs and PAC.

We used intraoperative HFOs and PAC to demonstrate the effectiveness of subtotal hemispherotomy as a treatment for ESs in a subset of patients with AS. The thalamocortical and subcortical pathways play a role in the ES network.

Written informed consent was obtained from the infant's parents. The ethics committee of Juntendo University (Tokyo, Japan) approved this study (No. 16-163).

## Data Availability Statement

The original contributions presented in the study are included in the article/supplementary material, further inquiries can be directed to the corresponding author/s.

## Ethics Statement

The ethics committee of Juntendo University (Tokyo, Japan) approved this study (No. 16-163). Written informed consent to participate in this study was provided by the participants' legal guardian/next of kin.

## Author Contributions

All authors listed have made a substantial, direct and intellectual contribution to the work, and approved it for publication.

## Conflict of Interest

The authors declare that the research was conducted in the absence of any commercial or financial relationships that could be construed as a potential conflict of interest.
